# A Novel Approach on the Use of Samples from Faecal Occult Blood Screening Kits for Metabolomics Analysis: Application in Colorectal Cancer Population

**DOI:** 10.3390/metabo13030321

**Published:** 2023-02-21

**Authors:** Oihane E. Albóniga, Joaquín Cubiella, Luis Bujanda, María Encarnación Blanco, Borja Lanza, Cristina Alonso, Beatriz Nafría, Juan Manuel Falcón-Pérez

**Affiliations:** 1 Metabolomics Platform, CICbioGUNE-BRTA, Centro de Investigación Biomédica en Red de Enfermedades Hepáticas y Digestivas (CIBERehd), Bizkaia Technology Park, 48160 Derio, Spain; 2 Department of Gastroenterology, Instituto de Investigación Sanitaria Galicia Sur, Centro de Investigación Biomédica en Red de Enfermedades Hepáticas y Digestivas (CIBERehd), Complexo Hospitalario Universitario de Ourense, 32005 Ourense, Spain; 3 Department of Gastroenterology, Centro de Investigación Biomédica en Red de Enfermedades Hepáticas y Digestivas (CIBERehd), Biodonostia Health Research Institute, Donosti Hospital, University of the Basque Country (UPV/EHU), 20014 San Sebastián, Spain; 4 OWL Metabolomics, Bizkaia Technology Park, 48160 Derio, Spain; 5 Clinical Biochemistry Department, Biodonostia Health Research Institute Osakidetza Basque Health Service, Donostialdea Integrated Health Organisation, 20014 San Sebastian, Spain; 6 Exosomes Laboratory, CIC bioGUNE-BRTA, Centro de Investigación Biomédica en Red de Enfermedades Hepáticas y Digestivas (CIBERehd), Bizkaia Technology Park, 48160 Derio, Spain; 7 IKERBASQUE, Basque Foundation for Science, 48011 Bilbao, Spain

**Keywords:** cholesteryl esters, colorectal cancer, semitargeted metabolomics, lipids, FOB screening kits

## Abstract

The incidence of colorectal cancer (CRC) is increasing, and currently it is the third most common cancer. Early CRC diagnosis is still difficult and relies on an invasive colonoscopy and tissue biopsy. The globally observed tendency demands non-invasive, specific, and accurate diagnostic tools for early diagnosis and prognosis. In this work, the main aim was to evaluate for the first time the feasibility of using extracts from the non-invasive sample collection from faecal occult blood (FOB) kits for its use in metabolomics studies taking advantage in this way of the high sensitivity of this technology. Then, a cohort of 131 samples from control individuals (CTL), adenoma (AD) and CRC patients were analysed using a semitargeted approach by ultra-high-performance liquid chromatography–time-of-flight–mass spectrometry (UHPLC-ToF-MS). Multivariate and univariate statistical analysis revealed that cholesteryl esters (ChoE) with polyunsaturated fatty acids (PUFAs) together with FOB were relevant metabolites that could clearly separate CRC patients from AD and CTL individuals, whereas the metabolic profiles of CTL and AD were very similar. These results are in agreement with previous findings and reveal the advantage of using the same FOBT samples for several analyses, which would facilitate sample collection and improve direct connection between FOB measurements and metabolomics analysis. Although the sample size and the number of metabolites should be enhanced to cover a wider range of metabolites, alterations in lipid metabolism clearly point out for future perspectives.

## 1. Introduction

The incidence of colorectal cancer (CRC) has increased globally and is currently the third most common cancer in the world (GLOBOCAN, 2020), accounting for 10% of all cancer deaths [1,2]. Most sporadic CRCs are developed through the formation of polypoid adenomas and are preceded by intramucosal carcinoma (high-grade dysplastic adenoma), which can progress into malignant forms [3]. This process is known as the adenoma–carcinoma sequence, and it takes decades before final malignancies develop [4]. Although it progresses slowly over a long period of time, the majority of patients are diagnosed at advanced stages, when prognosis is poor [5]. Treatment in the early stages of the disease has been reported to have a high 5-year survival rate (80–90%) compared with cases in which the tumour has metastasized [6]. The gold standards for screening and detection of CRC are colonoscopy and tissue biopsy, which are invasive and pose potential risks for complications, particularly if performed on elderly or seriously ill patients [2]. Therefore, the non-invasive faecal occult blood test (FOBT) and faecal immunochemical test (FIT) are the most popular screening methods, but exhibit low sensitivity [7,8], particularly in detecting early stages of the disease. For these reasons, and the need for screening methods that are non-invasive, specific and accurate in the early identification of CRC, have spurred several researchers to turn to the use of molecular techniques such as genomics, transcriptomics, proteomics and metabolomics to identify biomarkers [6]. Each science offers its own advantages for the discovery of cancer biomarker. In the particular case of metabolomics, which is the “omic” science closest to phenotype and dedicated to the measurement of metabolites presented in a biological system, has been widely employed for biomarkers identification of a multitude of diseases including CRC [7,9,10].

The most readily accessible bio-samples, such as faeces, urine, and blood, have great potential for the discovery of early cancer biomarkers or even precursors, such as adenomas, with minimally invasive sample collection. In this context, most studies of CRC using metabolomics have been performed in plasma, serum or urine samples, as previously reviewed [7,9], while far fewer have been conducted on faeces samples [9,11,12,13,14,15,16,17,18,19,20,21,22]. Metabolomics study of faeces may be more effective at detecting novel colon cancer markers because faeces are close to the colorectal mucosa. Previous research work has studied endogenous metabolic profiles differences of faeces from patients with adenoma and CRC, controlling patients with the use of targeted metabolomics [14]. This study proposed a robust metabolic signature of several metabolites as faecal biomarkers. The seven highlighted metabolites belonged to cholesteryl esters (ChoE), phosphatidylethanolamines (PE), sphingomyelins (SM) and triglycerides (TG). The analysis of ChoE (18:1), ChoE (18:2), ChoE (20:4), PE (16:0/18:1), SM (d18:1/23:0), SM (42:3) and TG (54:1) in faecal samples provided a non-invasive diagnostic tool for colon cancer population screening that was better than faecal occult blood (FOB) in the detection of CRC [14]. The results also pointed out that the combination of FOB with the seven metabolites of the metabolomic model increased the discriminatory ability for CRC patients. In this sense, it seems that ChoE should be considered a relevant lipidic family for the study of CRC.

In this study, considering previous findings as well as the importance of the highlighted metabolites, a semitargeted approach using ultra-high-performance liquid chromatography–mass spectrometry (UHPLC-MS) was used to determine not only ChoE (18:1), ChoE (18:2), ChoE (20:4), PE (16:0/18:1), SM (d18:1/23:0), SM (42:3) and TG (54:1), but also other cholesteryl esters such as ChoE (16:0), ChoE (18:3), ChoE (20:2), ChoE (20:5), ChoE (22:4), ChoE (22:5), and ChoE (22:6). Furthermore, bearing in mind the risk associated with CRC screening and diagnosis using biopsy or colonoscopy, and the lack of sensitivity of FOB and FOBT tests [8], an extraction procedure for metabolites in extracts obtained from the non-invasive kits used for FOBT was evaluated. In this sense, and to the best of our knowledge, this is the first research to study the feasibility use of extracts from FOBT to determine the potential differences among healthy individuals, patients with adenomas and patients with colorectal cancer using metabolomics.

## 2. Materials and Methods

### 2.1. Chemicals

HPLC-MS-grade solvents were purchased from Fisher Scientific (Hampton, NH, USA). Reference metabolite standard compounds were obtained from Sigma Aldrich (St. Louis, MO, USA), Larodan Fine Chemicals (Malmö, Sweden) and Avanti Polar Lipids (Alabaster, AL, USA).

### 2.2. Clinical Samples and Study Population

Samples were collected from the Biodonostia Health Research Institute (Gipuzkoa, Spain). The inclusion criteria were asymptomatic individuals with intermediate risk, that is, individuals from 50 to 69 years that do not have clinical symptoms, and patients that do not have any family history or other factor associated with colorectal cancer. The study was conducted according to the clinical and ethical principles of the Spanish Government and the Declaration of Helsinki, and was approved by the Ethics Committee for Clinical Research of Euskadi (PI2013073). Informed consent was obtained from each individual.

Patients and controls self-collected a faecal sample from one bowel movement without specific diet or medication restrictions at home the week before a colonoscopy was performed [23]. The faecal sample was delivered to the hospital and brought to the laboratory in less than 4 h, split in aliquots and immediately frozen at −80 °C [14]. One aliquot of each sample was employed for faecal occult blood (FOB) measurement. The remaining of each of the FOB extracts was frozen at −80 °C until used for metabolomics analysis.

Three different batches of samples were used. Samples were classified according to the batch and experimental group. Sample information is summarized in Table 1 and more detailed information is collected in Table S1. All batches, provided by Biodonostia Health Research Institute (Gipuzkoa, Spain) were collected with OC Sensor faecal immunochemical test (Eiken Chemical Co., Ltd., Tokyo, Japan). Among different batches, the first batch was used for extraction procedure evaluation, consisted of three samples in each group. A pool of each three samples was prepared and five replicates were performed from each pool.

### 2.3. Sample Collection and Metabolite Extraction

**Figure 1:**
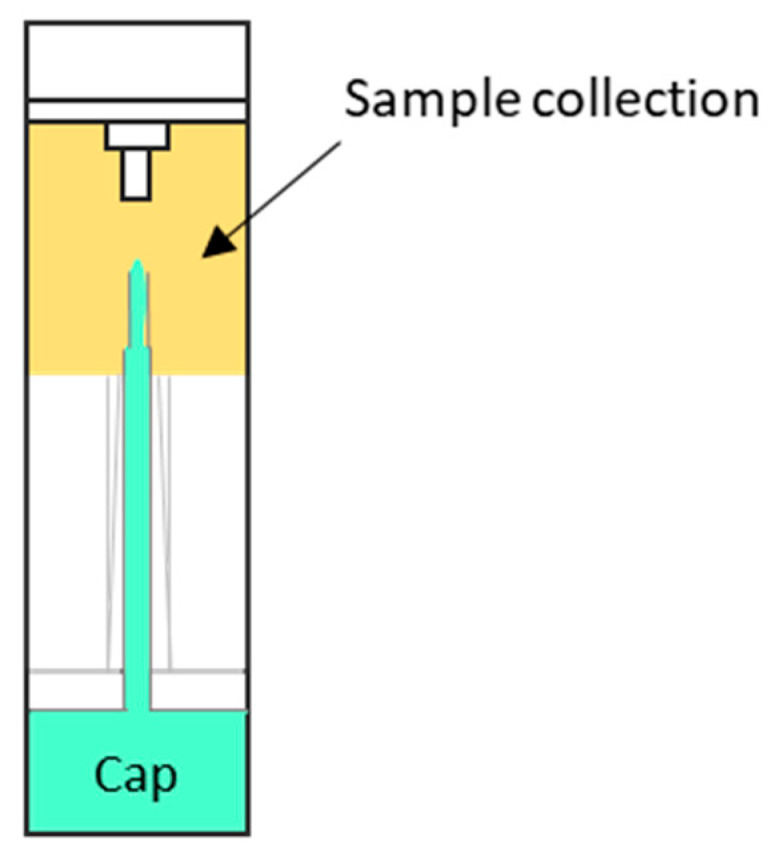
Image of OC-Auto sampling bottle for faecal immunochemical test (Eiken Chemical Co., Ltd., Tokyo, Japan. The black arrow indicates the sample collection point. Dimensions 2.0 × 8.0 cm.

Samples were thawed to room temperature on ice. Then, samples were extracted from the device by unscrewing the cap (Figure 1), squeezing the device, and collecting the sample in an Eppendorf microtube. Afterwards, 500 µL of samples for metabolite extraction and analysis were homogenized using a Precellys 24 homogenizer (Bertin Technologies, Montigny-le-Bretonneux, France) at 6500 rpm for 23 s. Then, 200 µL of homogenized faeces extracts were collected and transferred to 1.5 mL microtubes. Afterwards, 780 µL of chloroform:methanol (CHCl_3_:MeOH; 2:1) was added. The CHCl_3_:MeOH used for extraction was spiked with metabolites not detected in unspiked faeces extracts as internal standards (IS) [SM (d18:1/16:0), PE (17:0/17:0), PC (19:0/19:0), TAG (13:0/13:0/13:0), Cer (d18:1/17:0), and ChoE (12:0)]. After brief vortex mixing, samples were incubated for 1 h at −20 °C. After centrifugation at 18,000× *g* for 5 min, 600 µL of the organic phase were collected, dried under vacuum, and reconstituted in 100 µL acetonitrile:isopropanol (ACN:IPA; 1:1), centrifuged (18,000× *g* for 10 min), and transferred to vials for UHPLC-MS analysis.

Additionally, different types of quality control (QC) samples were used to assess data quality. Considering that response changes are particularly important at large scale, a QC calibration serum sample (QC_cal) was prepared to correct biases between and within batches. Additionally, a QC validation sample (QC_val) was prepared by pooling all samples within each batch to assess how well data pre-processing procedure improved the data quality. Both, QC calibration and QC validation samples were extracted and analysed at the same time as the individual samples in each analytical sequence. Two types of blanks were prepared. The QC blank sample was prepared following the same extraction procedure as biological samples, and the QC system suitability blank sample was prepared with the solvents used for biological samples reconstitution. Both blanks were included and analysed.

For the analytical sequence, randomized sample injections were performed with each of the QCs (QC_cal and QC_val) extracts uniformly interspersed throughout the entire batch run.

### 2.4. UHPLC-MS Metabolic Profiling

Metabolomics analysis of faeces extracts was performed using an ACQUITY UPLC system coupled to a QTOF Premier mass spectrometer, both from Waters (Milford, MA, USA), and equipped with an electrospray ionization source operating in positive ionization mode (ESI+). Chromatographic separation was carried out injecting 2 µL of sample on an ACQUITY UPLC BEH C18 column (1.0 × 100 mm, 1.7 µm), at 60 °C and a flow rate of 0.15 mL/min. A binary solvent system consisting of H_2_O:ACN (2:3) with 10 mM ammonium formate (solvent A) and ACN:IPA (1:3) with 10 mM ammonium formate (solvent B) was used for the elution. The gradient started from 60% A and 40% B, with a 10-min linear gradient increasing from 40% to 100% B. After 5 min at 100% B, the mobile phase was reset to the initial composition in readiness for the subsequent injection to a total run time of 17 min. The mass spectra data were acquired in positive ionization mode with capillary and cone voltages of 2000 and 35 V, respectively. The desolvation gas was set to 1000 L/h at a temperature of 400 °C. The cone gas was set to 30 L/h, and the source temperature was set to 120 °C. The MS detector operated in centroid acquisition mode for a *m*/*z* range of 50–1200, using an accumulation time of 0.5 s per spectrum. MS ion optics were tuned to a resolution of 20,000 FWHM which corresponded to a mass accuracy of 5 ppm. All spectra were mass corrected in real time by reference to leucine enkephaline, infused at 10 µL/min through an independent reference electrospray, sampled every 10 s. The overall quality of the analysis procedure was monitored using repeat extracts of the QC samples.

### 2.5. Data Pre-Processing

All data were processed using TargetLynx application manager for MassLynx 4.1 software (Waters Corp., Mildfor, CT, USA). A set of predefined retention time–mass-to-charge ratio pairs, RT-*m*/*z*, corresponding to metabolites included in the analysis, and identified based on an in-house library with a mass tolerance window of 0.05 Da, were fed into the program. Associated extracted ion chromatograms (EICs; mass tolerance window = 0.05 Da) were then peak-detected and noise-reduced in both the LC and MS domain such that only true metabolite-related features were processed by the software. A list of chromatographic peak areas was then generated for each sample injection.

For identified metabolites, representative MS detection response curves were generated using an internal standard for each chemical class included in the analysis. By assuming similar detector response levels for all metabolites belonging to a given chemical class represented by a single standard compound, a linear detection range was defined for each identified metabolite. Maximum values were defined as those at which the detector response became non-linear with respect to the concentration of the representative internal standard. Variables not considered for further analysis, where more than 30% of data points were found outside their corresponding linear detection range, were removed.

### 2.6. Data Normalization and Quality Control

Instrumental drifts in MS-driven metabolomics analysis were taken into consideration; an intra- and inter-batch normalization based on multiple internal standards and pool calibration samples approach were used. Following the procedure described previously, the intra-batch normalization was implemented using multiple internal standards response correction [24]. Thus, the most appropriate internal standard for each variable was defined as that which resulted in a minimum relative standard deviation after correction, as calculated from the QC calibration samples over all the analysis batches. Once the internal standard correction had been carried out, a possible random or drift distribution in the QC calibration samples along the batch was determined for each variable. For this, robust linear regression (internal standard corrected response as a function of sample injection order) was used to estimate in the QC calibration samples any intra-batch drift not corrected by internal standard correction. For all variables, internal standard corrected response in each batch was divided by its corresponding intra-batch drift trend, such that normalized abundance values of the study samples were expressed with respect to the batch averaged QC calibration serum sample (arbitrary set to 1). Finally, the assessment of reproducibility was calculated using the QC validation samples of each batch. Any remaining sample injection variable response zero values in the corrected dataset (missing values) were imputed using *k*-nearest neighbour algorithm (*k*NN) before generating the final dataset that was used for study sample statistical analysis.

### 2.7. Statistical Analysis

Once data had been normalized by each appropriate IS and trend had been corrected, the percentage of coefficient of variance (% CV) was calculated to determine each metabolite’s reproducibility. To do this, the standard deviation was divided by the average in the QC sample and multiplied by one hundred before any statistical analysis. Then, statistical analysis was performed following two complementary approaches: multivariate and univariate analysis. The first step in multivariate data analysis was to reduce the dimensionality of the complex data set to enable easy visualization of any clustering of the different groups of samples as well as to detect outliers. This was achieved by principal component analysis (PCA), where the data matrix is reduced to a series of latent variables or principal components (PCs). Different labels were used for tendency grouping such as age, gender, FOB and disease stage. Then, supervised methods, such as partial least squares discriminant analysis (PLS-DA) and orthogonal PLS-DA (OPLS-DA) were used for classification and variable selection after an appropriate model validation. Afterwards, univariate statistical analysis was performed using a parametric or non-parametric approach depending on the normality test results.

Firstly, normality was tested by using the Kolmogorov–Smirnov–Lilliefors (KSL) test, followed by one-way ANOVA or Kruskal–Wallis to determine significant metabolites among the groups under study (CTL, AD and CRC) with *p*-value ≤ 0.05. Afterwards, an unpaired *t*-test or Mann–Whitney U test was performed for the following two-by-two group comparison: (1) AD vs. CTRL; (2) CRC vs. CTRL, and (3) CRC vs. AD. In all cases, to control the false discovery rate (FDR), *q*-values were generated using the Benjamini–Hochberg approach (*q*-value ≤ 0.05). Finally, log_2_ (fold-change) was also calculated for each two-by-two comparison. All the statistics were performed using the Umetrics SIMCA-P software version 13.0.1 (Umetrics, Umea, Sweden) and MATLAB software (The MathWorks, Naticks, MA, USA).

## 3. Results

### 3.1. Reproducibility of Metabolite Extraction Procedure (Batch 1)

Five replicates of the pooled samples prepared for each group from Batch 1 (pool CTL, pool AD, and pool CRC) were used to determine the CV percentage of each metabolite as it was previously mentioned. Due to two QC sample types being analysed throughout the sequence (QC_val and QC_cal), two normalizations could be performed. In this sense, both QCs were analysed to determine % CV and reproducibility. The % CV values are included in Tables S2 and S3 for QC_val and QC_cal, respectively. Considering that for metabolomics studies a % CV range from 20 to 30 [25], and for biomarkers an upper limit of 30% [26], is considered acceptable, it can be seen from Tables S2 and S3 that all metabolites have CV less than 30% except for ChoE (20:4) for pooled CRC. This might be due to the concentration in the pool sample being close to the detection limit. It should be noted that in this first batch, only the metabolites reported in previous studies were included in order to assess reproducibility, and then, for further batches, the 14 metabolites were analysed. Finally, as mentioned previously, normalization using QC_cal was performed, since this made it possible to gather these data and compare them with future data generated from samples collected in other studies. As can be seen from Table S3, and in comparison with Table S2, the % CV of each metabolite differs slightly. Furthermore, analysing the QC_val results (Table S3), it can be seen that all % CV values are lower than 8%, indicating a good reproducibility of the chromatographic and detection method.

### 3.2. Metabolic Differences per Group

Having evaluated the reproducibility of the metabolites’ extraction procedure and the chromatographic and detection methods, 33 and 98 human faecal extracts from Batch 2 and Batch 3, respectively, were analysed. All metabolites had % CV less than 30% in QCval2 and QCval3 and total ion chromatograms were perfectly overlapped (data not shown).

Once data quality had been checked, normalized and trend corrected using the QC_cal sample as previously mentioned, multivariate statistical analysis was performed in Batch 2 and Batch 3. As the results obtained were very similar in both batches, with slight tendencies observed in PCAs and no validated PLS-DA or OPLS-DA models, only results from Batch 2 were included in the Supplementary Materials as examples of multivariate statistical workflow. As can be seen in the PCA scores plot (Figure S1), CRC samples were mainly located in the right upper part of the scores plot. Furthermore, each sample was labelled by gender and by FOB to determine if sex or the amount of FOB had a greater influence in separation than the disease itself. Age was not included due to groups having same age range, except for a CTL individual with 24 years of age (Table 1) that was excluded from the analysis. As can be observed from PCA scores plots, no separation tendency was found based on gender (Figure S2) or FOB (Figure S3) and thus, these variables did not influence the tendency observed among groups and was not correlated with disease stage.

A supervised model was then generated to include the information related to each class. In this sense, the PLS-DA increased the separation tendency of CRC compared to AD patients and CTL individuals. Even though a clear tendency was shown in the PLS-DA scores plot (Figure S4), the model did not pass any of the validation criteria. Thus, two-by-two OPLS-DA models were built. The two OPLS-DA models obtained are included in Supplementary Materials (Figures S5 and S6), and included CTL vs. CRC and AD vs. CRC samples of Batch 2. OPLS-DA scores plots were generated and showed a good separation between groups; however, the model did not pass any of the validation criteria (CV-ANOVA *p*-value ≤ 0.05, leave-one-out cross validation (LOOCV) or permutation testing), so it could not be used for variable selection. However, Variable Importance on Projection (VIP > 1) was checked to determine which variables influenced more in the models. For CTL vs. CRC comparison, ChoE (20:4), ChoE (18:2), ChoE (18:1), and ChoE (20:5) influence more on group separation. Additionally, FOB was highly influential, even though it was not sufficient for group separation as a unique variable. All these variables had VIP values greater than 1 and fulfilled the jack knife criteria. In the case of AD vs. CRC, the variables with the greatest influence on group separation were ChoE (20:4), ChoE (18:2) and FOB. Even though model validation was not obtained, several interesting variables were identified for tentative group separation. In the case of Batch 3, as only two samples were CRC, no separation was obtained between AD and CTL. This lack of discrimination between those two groups have been previously reported [14], indicating that metabolic signature of ChoEs are not sufficient to clearly distinguish AD and CTL individuals.

A supervised model was then generated to include the information related to each class. In this sense, the PLS-DA increased the separation tendency of CRC compared to AD patients and CTL individuals. Even though a clear tendency was shown in the PLS-DA scores plot (Figure S4), the model did not pass any of the validation criteria. Thus, two-by-two OPLS-DA models were built. The two OPLS-DA models obtained are included in Supplementary Materials (Figures S5 and S6), and included CTL vs. CRC and AD vs. CRC samples of Batch 2. OPLS-DA scores plots were generated and showed a good separation between groups; however, the model did not pass any of the validation criteria (CV-ANOVA *p*-value ≤ 0.05, leave-one-out cross validation (LOOCV) or permutation testing), so it could not be used for variable selection. However, Variable Importance on Projection (VIP > 1) was checked to determine which variables influenced more in the models. For CTL vs. CRC comparison, ChoE (20:4), ChoE (18:2), ChoE (18:1), and ChoE (20:5) influence more on group separation. Additionally, FOB was highly influential, even though it was not sufficient for group separation as a unique variable. All these variables had VIP values greater than 1 and fulfilled the jack knife criteria. In the case of AD vs. CRC, the variables with the greatest influence on group separation were ChoE (20:4), ChoE (18:2) and FOB. Even though model validation was not obtained, several interesting variables were identified for tentative group separation. In the case of Batch 3, as only two samples were CRC, no separation was obtained between AD and CTL. This lack of discrimination between those two groups have been previously reported [14], indicating that metabolic signature of ChoEs are not sufficient to clearly distinguish AD and CTL individuals.

Complementary to multivariate analysis, univariate statistical analysis was performed for the 14 metabolites as well as for FOB included in the study to determine potential metabolic differences among the group of samples in Batch 2 and Batch 3 separately. As previously mentioned, the KSL test was applied and then one-way ANOVA or Kruskal–Wallis was performed (see Table S4, sheets Batch 2 and Batch 3). In total, only nine metabolites in Batch 2 (Table S4, sheet Batch 2) fulfilled the requirements of the normality test; however, no significant results were obtained by one-way ANOVA. In the remaining metabolites, Kruskal–Wallis was applied and only FOB was significant among the three groups (*q*-value ≤ 0.05) (see Table S4, sheets Batch 2 and Batch 3). As mentioned previously, a multicomparison test was then applied and significant differences were obtained only between CTL and CRC or AD and CRC groups, but not between CTL and AD. For this reason, two-by-two analysis was performed and the results are collected in Table 2 and Table 3 for Batch 2 and Batch 3, respectively. Both tables display the results for *q*-value of unpaired *t*-test or Mann–Whitney U test, together with the log_2_(fold-change) for the comparisons AD vs. CTL, CRC vs. CTL, and CRC vs. AD. Considering that Batch 3 only contained two samples in the CRC group, only CTL and AD were compared. Those significant metabolites (*q*-value < 0.05) are shaded in green. The log_2_(FC) is highlighted in red (highly upregulated with log_2_(FC) values greater or close to 1), when, for example, CRC patients had more than double the abundance of ChoE (18:2) found in AD patients. Similarly, but conversely, downregulated tendency is highlighted in blue (highly downregulated with log_2_(FC) values greater or close to −1). In this case, SM (d18:1/23:0) was found to be downregulated in the AD group compared to the CTL group, which means that this metabolite had double the abundance in the CTL group that it did in AD patients.

No significant differences have been observed between CTL and AD groups in any studies (Table 2 and Table 3). On the remaining two comparisons, ChoE (20:4) (*q*-value = 0.0473) and FOB (*q*-value = 0.0012) were significant when CRC was compared with CTL, and ChoE (20:4) (*q*-value = 0.0473), ChoE (18:2) (*q*-value = 0.0385), and FOB (*q*-value = 0.0186) were significant when CRC was compared with AD. Furthermore, we considered it to be of relative importance that several ChoEs had log_2_(FC) ≥ 1, which means more than double the abundance in CRC compared to in CTL or AD individuals, and log_2_(FC) ≤ 1, which means that CRC patients had abundances that were half those in CTL or AD. It is also remarkable that most of the highlighted ChoEs have polyunsaturated fatty acids (PUFAs) linked to the cholesterol molecule.

Analysing CTL and AD and comparing them, no significant differences have been observed in any study (Table 2 and Table 3). Despite this fact, the log_2_(FC) observed for ChoE (22:6) and ChoE (20:2) in Batch 2 and Batch 3, respectively, followed an upregulated tendency with more than double abundances in AD patients. Conversely, SM (d18:1/23:0), and TG (54:1) followed a downregulated tendency with important differences in abundance between AD and CTL individuals. These last tendencies were not observed with such differences in Batch 3, but we consider them to be of special interest for future studies on non-targeted approaches covering a wider range of metabolites and lipids. It might also be pointed out that these findings and tendencies are in agreement with previously published work in which it was found that cholesteryl esters class was consistently increased in cancer samples [14]. Thus, even though statistical significance was not achieved, the observed tendency and the dysregulation between groups were consistent with other studies.

Analysing the results, and comparing them with previous findings [14], ChoE (20:4) was found to be upregulated in all patients (AD and CRC) compared to healthy individuals (CTL), which is in agreement with the results obtained in batches 2 and 3. Thus, the significant metabolite ChoE (20:4) might be a potentially interesting biomarker due to the dysregulation between groups, but it should be further studied with a bigger sample size and specific method in order to extract more reliable conclusions. Additionally, FOB is a valuable variable that should be included for CRC diagnosis to complete other measurements as it influenced on group separation in combination with other metabolites but not alone. Finally, and as can be observed in Table 2 and Table 3, a disagreement regarding tendency is found in SMs. In Batch 2, SM (d18:1/23:0) is upregulated compared to the downregulated tendency found in Batch 3 for CTL vs. AD individuals. Same behaviour, but the opposite is observed for SM (42:3). In these cases, SM (d18:1/23:0) in Batch 2 and SM (42:3) in Batch 3 are in agreement with the upregulated tendency observed in AD patients compared to healthy individuals [14]. Further studies are needed to clarify these findings as well as include other SMs in the analysis to enhance the coverage of this lipid class. Finally, the remaining metabolites, PE (16:0/18:1) and TG (54:1), were considered, but no consistent results could be obtained for this independent batches.

In order to obtain a more representative cohort from the general Spanish population, both batches were fused to enhance the number of samples per group.

### 3.3. Fusion of Independent Studies

Due to the normalization performed in separate batches being comparable, samples from Batch 2 and Batch 3 were fused. In total, 131 human faecal extracts were considered and classified as detailed in Table 4.

**Figure 2:**
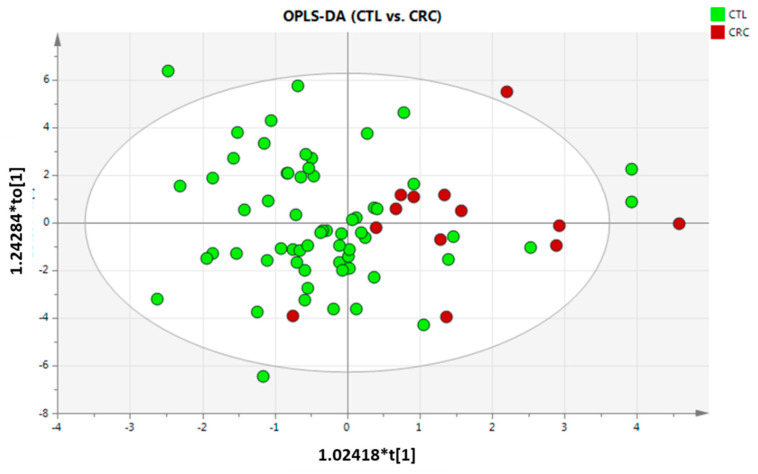
OPLS-DA scores plot obtained for the comparisons between CTL vs. CRC (R^2^ = 0.251, and Q^2^ = 0.0068; CV-ANOVA *p*-value = 0.9753). CTL—control; and CRC—colorectal cancer individuals. Scaling and transformation were autoscaled and logarithmic.

Following the same pipeline, PCA did not show any separation tendency or grouping (Figure S7), as also obtained in previous findings [11]. As done previously, PCA labelled by gender and FOB were built. No separation tendency was obtained by gender or FOB (data not shown). Afterwards, PLS-DA was built, but did not show any clustering (data not included), so two-by-two OPLS-DA was performed. Only CTL vs. CRC comparison generated an OPLS-DA model (Figure 2), but the model did not pass any validation criteria. Despite this fact, VIP values were evaluated, and it was found that FOB, SM (42:3), ChoE (20:4), ChoE (18:2) and ChoE (20:5) were those variables that most influence on group separation. The lack of model generation and validation might be due to CRC group only contain 13 individuals compared to the other two groups, which contain 62 and 56 for CTL and AD, respectively. CTL and AD groups had an appropriate sample size, but no separation was observed. This indicates that healthy individuals and patients with adenoma did not have enough differences in the abundance of the metabolites here included to clearly separate each other.

Afterwards, univariate statistical analysis was performed, similarly to in the previous independent batches. Only FOB was statistically significant when Kruskal–Wallis was applied; however, FOB only differentiates CTL and AD from CRC patients, but not CTL from AD (see Table S4, sheet Fused Batches). Due to any of the metabolites being statistically significant among the three groups (CTL, AD and CRC), two-by-two comparisons were performed. In Table 5, the *q*-values for AD vs. CTL, CRC vs. CTL and AD vs. CRC are gathered for the unpaired Mann–Withney U test and the log_2_(FC) of each metabolite. As previously explained, those significant metabolites (*q*-value < 0.05) are shaded in green, and the tendency is highlighted in red (highly upregulated with log_2_(FC) values greater or close to 1) and in blue (highly downregulated with log_2_(FC) values greater or close to −1).

As can be observed, FOB, ChoE (20:4), ChoE (18:2) and SM (42:3) were significant when CRC patients were compared to CTL individuals, doubling the abundances in CRC patients for the metabolites FOB, ChoE (20:4) and SM (42:3). Other ChoEs had log_2_(FC) greater than 1 and thus, even if no significant values were obtained, they should be considered for futures studies (Table 5). Considering the remaining comparisons, only ChoE (20:2) had abundances in AD patients that were double those in CTL individuals, and SM (42:3) was statistically significant, with double the abundance in CRC compared to AD. These results allowed us to see that there were metabolic differences between groups, but 14 metabolites are not sufficient for classification, and more lipid classes as well as an increment in sample size are needed.

## 4. Discussion

To the best of our knowledge, this study constitutes the first to evaluate the feasibility of remnants of faecal occult blood tests (FOBT) as samples for metabolomics studies by liquid chromatography coupled to mass spectrometry (LC-MS). Our study compares 14 metabolites analysed from the remnants of FOBT samples among three groups, and it was demonstrated that it is reproducible, and several metabolites can be measured. An untargeted metabolomics analysis would be a very interesting approach to complement this study and to cover a wider range of metabolites and better determine the feasibility of this biological matrix. We found of special importance the fact that FOB analysis, performed directly in the same samples used for metabolomics studies, influenced group separation. This was of tremendous relevance due to all variables and metabolites being measured in the same sample, thus preserving the integrity of both FOB analysis and metabolomics.

Focusing on the 14 analysed metabolites that were classified as different lipid classes, cholesteryl esters with polyunsaturated fatty acids (PUFAs) were those with highest differences between group abundances. Alterations in lipid metabolism are currently considered a characteristic feature of many malignancies, including CRC [27].

Evidence has been reported by several authors that CRC is associated with alterations in fatty acid profiles, in particular increased levels of saturated and monounsaturated very-long-chain FAs in tumour tissue and sera of CRC patients, co-existing with enhanced expression of FA elongases 1 and 6 in cancer tissue [28,29]. Serum PUFA content has to be incorporated in diet and/or supplementation for two of them, linoleic acid (LA, 18:2) and α-linolenic acid (ALA, 18:3), because humans do not possess enzymes required for LA and ALA synthesis [30]. Once delivered to the human body, LA and ALA can be metabolized to other PUFAs by omega-6 (ω-6) and omega-3 (ω-3) pathways, with LA being an essential precursor for ω-6, and ALA (18:3) for ω-3. Both pathways are intercorrelated with several desaturation and elongation reactions [31,32]. PUFAs have a huge number of functions in the human body, such as structural phospholipids of cell membranes, they modulate membrane fluidity, cellular signalling and cellular interaction. Apart from these, they play an extremely important role in the regulation of the immune system response by acting as precursors for the synthesis of eicosanoids. These metabolites are synthesized from the 20-carbon PUFA precursors [30]. In this sense, PUFAs may attenuate or enhance the inflammation process implicated in CRC development. Thus, ω-3 PUFAs produce anti-inflammatory effects, whereas ω-6 PUFAS, especially arachidonic acid (ARA, 20:4), are known as precursors of proinflammatory eicosanoids [28].

Other studies have assessed the relationship between ω-6 PUFAs and CRC, which showed that high dietary intake and plasma levels of ω-6 PUFAs might act as a tumour promoter and increase the risk of CRC [15,33,34]. Song et al. [15] analysed faecal metabolomes and concluded that ω-6 PUFAs could be risk factors for CRC development, while a high dietary intake also increased faecal long-chain ω-6 PUFAs. They hypothesized that an altered faecal level of long-chain PUFAs may influence the pathogenesis of CRC through two processes. The first indicated the influence on faecal PUFA level by dietary intake of PUFAs that could be related to the level of plasma lipids through systemic absorption, thereby affecting CRC development via a systemic effect. The second is related to the changes in faecal fatty acids that may influence the structure and function of the colonic mucosa via direct contact [15].

ChoEs, the main lipid class in this study, are structurally composed of a cholesterol linked to a fatty acid by an ester bond. The fatty acid can be of different length and saturation level. In our particular case, the significant or relevant ChoEs in CRC contain long-chain FA with two or more double bonds, which means that cholesterol is linked to a PUFA. In this way, ChoE (18:2), ChoE (20:2), ChoE (20:4), and ChoE (22:5) contain PUFAs that belong to ω-6 pathway, and ChoE (20:5), and ChoE (22:6) to ω-3.

It was found in mice that the concentration of cholesteryl ester in the liver varied markedly in the different diet groups (FA 18:0, FA 14:0, FA 18:1 and FA 18:2), even though hepatic cholesterol balance in these animals was not different [35]. They concluded that these variations reflected differences in the ability of the specific fatty acids to drive equilibrium of the enzyme acyl-coenzyme A:cholesterol acyltransferase (ACAT) reaction in the direction of esterification. They saw that enriching the liver with either the FA 18:1 or 18:2 increased hepatic cholesteryl ester 6-fold, and that dietary FAs differentially regulated the steady-state level of ChoE in the liver, dictating the rate of sterol incorporation into very low density lipoprotein (VLDL) particles and secretion into the plasma [35].

Finally, Cubiella et al. identified and analysed the expression levels of gene-encoding proteins involved in glycerophospholipids, and sphingolipids metabolism, and glycosylphosphatidylinositol (GPI)-anchor biosynthesis pathway, and they observed that the gene related to LCAT was upregulated in patients with CRC [14]. This gene encodes an enzyme involved in the synthesis of cholesteryl ester.

Taking into account all these facts, it might be possible that a high dietary intake containing PUFAs, or the availability of PUFAs associated with colorectal cancer hypothesized by Song et al. [15], together with the upregulation on LCAT gene, could also be associated with an increment in ChoE synthesis that at the same time could be related with those higher levels of PUFAs observed previously in CRC patients [15]. In order to better explain this phenomenon, free PUFAs and LCAT activity as well as other lipids should be measured to shed more light on the pathophysiological mechanism behind CRC.

## 5. Conclusions

Sampling from remnant FOB test has several advantages, such as the fact that it can be easily collected from each individual, avoiding any invasive sample collection such as colonoscopy or biopsy, and the measurement of FOB can be compared directly with metabolomics results, as both analyses can be made from the same sample. All these points make this pilot study relevant for enhancing sample collection, making it completely non-invasive for future studies related to colorectal cancer. Furthermore, the possibility of using this biological matrix for metabolomics approaches to determine differences among groups has been demonstrated. In this case, semi-targeted metabolomics was applied to determine several lipids, mainly cholesteryl esters, in a Spanish population that had been diagnosed as being adenoma or colorectal cancer patients or healthy individuals. We found some differences in tendencies in cholesteryl esters composition between at least two groups that is consistent with previous research findings.

Apart from these relevant points, this pilot study has several limitations that should be considered, such as the small number of enrolled patients, the heterogeneity among individuals, and the lack of information about the dietary habits of the patients, mainly assumed to follow a Mediterranean diet. As it seems that dietary intake could be related to PUFA abundances, and consequently to the alteration in lipids classes with esterified long-chain PUFAs, more systematic studies are necessary to better control dietary habits. This makes the results and conclusions of our study tentative findings. Finally, our current research investigated the results of certain metabolites or important lipid classes previously highlighted as putative biomarkers but not global metabolites. In this sense, a wider range of metabolites should be comprehensively included in future studies to determine group differences.

In summary, our current study showed for the first time that remnant samples from FOBT kits can be used for metabolomics analysis. Changes in measured metabolites were observed between at least two groups, those being cholesteryl esters with long-chain polyunsaturated fatty acids the most altered metabolites. Larger profiling studies based on lipidomics approach and polar compounds are needed to evaluate patients and control individuals.

## Figures and Tables

**Table 1:** Sample classification.

Batch	Experimental Group	Group Code	Number of Samples	Gender (% Women)	Age, Median
Batch 1	Control	CTL	3	NA ^1^	NA ^1^
	Adenoma	AD	3	NA ^1^	NA ^1^
	Colorectal cancer	CRC	3	NA ^1^	NA ^1^
Batch 2	Control	CTL	11	54	61.5 (24–70) ^2^
	Adenoma	AD	11	18	60 (53–64) ^2^
	Colorectal cancer	CRC	11	45	62 (50–69) ^2^
Batch 3	Control	CTL	51	55	61 (51–70) ^2^
	Adenoma	AD	45	47	63 (50–71) ^2^
	Colorectal cancer	CRC	2	50	70 (69–71) ^2^
Batch 2 + 3	Control	CTL	62	41	61 (24–70) ^2^
	Adenoma	AD	56	56	63 (50–71) ^2^
	Colorectal cancer	CRC	13	46	65 (50–71) ^2^

^1^ NA: not applicable. ^2^ The age in brackets refers to the minimum and maximum ages of each group.

**Table 2:** Log_2_(fold-change) and *q*-values of unpaired Mann–Whitney U test in the two-by-two comparisons in Batch 2.

		Adenoma vs. Colorectal Cancer (AD vs. CTL)	Colorectal Cancer vs. Control (CRC vs. CTL)	Colorectal Cancer vs. Adenoma (CRC vs. AD)
FOB	*q*-value	1	0.0012	0.0186
	log_2_(FC)	−0.6748	1.6339	2.3088
ChoE (16:0)	*q*-value	1	1	1
	log_2_(FC)	0.0791	−0.0182	−0.0973
ChoE (18:1)	*q*-value	0.3990	0.1419	1
	log_2_(FC)	−0.1491	−0.1936	−0.04453
ChoE (18:2)	*q*-value	1	0.3042	0.0385
	log_2_(FC)	−0.7916	0.3736	1.1653
ChoE (18:3)	*q*-value	1	1	1
	log_2_(FC)	−0.2334	−0.1391	0.09433
ChoE (20:2)	*q*-value	1	1	1
	log_2_(FC)	0.6879	−0.1814	−0.8693
ChoE (20:4)	*q*-value	1	0.0473	0.0473
	log_2_(FC)	−0.7378	1.3763	2.1141
ChoE (20:5)	*q*-value	1	1	1
	log_2_(FC)	0.3711	2.2539	1.8828
ChoE (22:4)	*q*-value	1	1	0.6508
	log_2_(FC)	−0.3206	−0.6638	−0.3432
ChoE (22:5)	*q*-value	1	1	1
	log_2_(FC)	−0.5777	1.2950	1.8732
ChoE (22:6)	*q*-value	1	0.8109	1
	log_2_(FC)	1.1112	1.6021	0.4909
PE (16:0/18:1)	*q*-value ^1^	0.7886	1	0.4757
	log_2_(FC)	0.5419	−0.1871	−0.7290
SM (d18:1/23:0)	*q*-value	1	1	1
	log_2_(FC)	−1.5919	−0.6195	0.9724
SM (42:3)	*q*-value	1	1	1
	log_2_(FC)	−0.5073	0.0852	0.5924
TG (54:1)	*q*-value	1	1	1
	log_2_(FC)	−1.1873	−2.9308	−1.7435

Note: green shading means significant metabolites (*q*-value < 0.05): red shading means upregulated and blue shading means downregulated tendency metabolite in more than double abundance in AD or CRC compared to CTL or AD groups. ^1^ *q*-value obtained from corrected *t*-test after normality was fulfilled.

**Table 3:** Log_2_(fold-change) and *q*-values of unpaired Mann–Whitney U test comparison in the two-by-two comparisons in Batch 3.

		Adenoma vs. Control (AD vs. CTL)
FOB	*q*-value	1
	log_2_(FC)	−0.3327
ChoE (16:0)	*q*-value	1
	log_2_(FC)	0.2828
ChoE (18:1)	*q*-value	0.8655
	log_2_(FC)	0.9083
ChoE (18:2)	*q*-value	1
	log_2_(FC)	0.5871
ChoE (18:3)	*q*-value	0.6151
	log_2_(FC)	0.7292
ChoE (20:2)	*q*-value	0.7600
	log_2_(FC)	1.0948
ChoE (20:4)	*q*-value	1
	log_2_(FC)	0.6010
ChoE (20:5)	*q*-value	1
	log_2_(FC)	0.4355
ChoE (22:4)	*q*-value	1
	log_2_(FC)	0.9314
ChoE (22:5)	*q*-value	0.9695
	log_2_(FC)	0.2337
ChoE (22:6)	*q*-value	1
	log_2_(FC)	0.3159
PE (16:0/18:1)	*q*-value	0.7418
	log_2_(FC)	0.0263
SM (d18:1/23:0)	*q*-value	1
	log_2_(FC)	0.0510
SM (42:3)	*q*-value	0.4398
	log_2_(FC)	0.3600
TG (54:1)	*q*-value	1
	log_2_(FC)	0.0009

Note: red shading means upregulated tendency metabolite in more than double abundance in AD compared to CTL group.

**Table 4:** Sample classification after fusing Batch 2 and Batch 3 populations.

Experimental Group	Group Code	Batch	Number of Samples	Total Number of Samples
Control	CTL	2	11	62
		3	51	
Adenoma	AD	2	11	56
		3	45	
Colorectal cancer	CRC	2	11	13
		3	2	

**Table 5:** Log_2_ (fold-change) and *q*-values of unpaired Mann–Whitney U test in the two-by-two comparisons in fused batches.

		AD vs. CTL	CRC vs. CTL	CRC vs. AD
FOB	*q*-value	1	0.0186	0.0012
	log_2_(FC)	−0.6749	1.6339	2.3088
ChoE (16:0)	*q*-value	1	1	1
	log_2_(FC)	0.2446	0.1119	−0.1327
ChoE (18:1)	*q*-value	0.3847	0.8639	1
	log_2_(FC)	0.7526	−0.0471	−0.7980
ChoE (18:2)	*q*-value	1	0.0384	0.1852
	log_2_(FC)	0.3965	0.4982	0.1017
ChoE (18:3)	*q*-value	0.8146	0.3290	1
	log_2_(FC)	0.5663	0.2705	−0.2958
ChoE (20:2)	*q*-value	0.6880	1	1
	log_2_(FC)	1.0329	0.9844	−0.0484
ChoE (20:4)	*q*-value	0.2518	0.0384	0.1329
	log_2_(FC)	0.4464	1.1898	0.7435
ChoE (20:5)	*q*-value	1	0.2294	0.8909
	log_2_(FC)	0.4231	2.0564	1.6333
ChoE (22:4)	*q*-value	1	1	0.9124
	log_2_(FC)	0.6970	0.1098	−0.5872
ChoE (22:5)	*q*-value	0.7798	1	1
	log_2_(FC)	0.1128	1.0324	0.9195
ChoE (22:6)	*q*-value	1	0.7371	1
	log_2_(FC)	0.4306	0.7851	0.3545
PE (16:0/18:1)	*q*-value	0.3456	1	1
	log_2_(FC)	0.0966	0.5939	0.4973
SM (d18:1/23:0)	*q*-value	1	0.4713	0.6079
	log_2_(FC)	−0.3906	0.3918	0.7824
SM (42:3)	*q*-value	0.6272	0.0112	0.0589
	log_2_(FC)	0.0754	1.1902	1.1148
TG (54:1)	*q*-value	1	1	1
	log_2_(FC)	−0.2906	−0.6918	−0.4012

Note: green shading means significant metabolites (*q*-value < 0.05): red shading means upregulated tendency metabolite in more than double abundance in AD or CRC compared to CTL or AD.

## Data Availability

The MS data are available at the NIH Common Fund’s National Metabolomics Data Repository (NMDR) website, the Metabolomics Workbench, https://www.metabolomicsworkbench.org (accessed on 2 February 2023), where it has been assigned project ID PR001594. The data can be accessed directly via its project DOI: “http://dx.doi.org/10.21228/M8NQ69”).

## Supplementary Materials

The following supporting information can be downloaded at: https://www.mdpi.com/article/10.3390/metabo13030321/s1, Figure S1: PCA-X scores plot obtained for the three studied groups (R^2^ = 0.612, and Q^2^ = 0.374) and coloured by class of Batch 2. CTL—control; AD—adenoma, and CRC—colorectal cancer individuals. Scaling and transformation were autoscaled and logarithm; Figure S2: PCA-X scores plot obtained for the three studied groups (R^2^ = 0.612, and Q^2^ = 0.374) and coloured by gender. 0—female, and 1—male individuals. Scaling and transformation were autoscaled and logarithm; Figure S3: PCA-X scores plot obtained for the three studied groups (R^2^ = 0.612, and Q^2^ = 0.374) and coloured by faecal occult blood. Scaling and transformation were autoscaled and logarithm; Figure S4: PLS-DA scores plot obtained for the three studied groups (R^2^ = 0.302, and Q^2^ = 0.149). CTL—control; AD—adenoma, and CRC—colorectal cancer individuals. Scaling and transformation were autoscaled and logarithm; Figure S5: OPLS-DA scores plot obtained for control individuals (CTL) and colorectal cancer patients (CRC) groups (R^2^ = 0.554, and Q^2^ = 0.276; CV-ANOVA *p*-value = 0.2431). CTL—control individuals, and CRC—colorectal cancer patients. Scaling and transformation were autoscaled and logarithm; Figure S6: OPLS-DA scores plot obtained for adenocarcinoma (AS) and colorectal cancer (CRC) groups (R^2^ = 0.675, and Q^2^ = 0.296; CV-ANOVA *p*-value = 0.1778). AD—adenoma, and CRC—colorectal cancer patients. Scaling and transformation were autoscaled and logarithm; Figure S7: PCA-X scores plot obtained for the three studied groups (R^2^ = 0.482, and Q^2^ = 0.23) when fusing Batch 2 and Batch 3 studies. CTL—control; AD—adenoma, and CRC—colorectal cancer individuals. Scaling and transformation were autoscaled and logarithm; Table S1: Sample information Metadata; Table S2: Percentage of coefficient of coefficient of variation (% CV) of each metabolite analysed, using a pool of samples as quality control validation sample (QCval_Rep). CTL: control or healthy individuals; AD: adenoma patients, and CRC: colorectal cancer patients; Table S3: Percentage of coefficient of coefficient of variation (% CV) of each metabolite analysed, using a commercial reference serum as quality control calibration sample (QC_cal). CTL: control or healthy individuals; AD: adenoma patients, and CRC: colorectal cancer patients; and Table S4: Statistical Analysis.
