# Supplementary material for: A Novel Approach on the Use of Samples from Faecal Occult Blood Screening Kits for Metabolomics Analysis: Application in Colorectal Cancer Population
Source: Metabolites. 2023 Feb 21;13(3):321. doi: 10.3390/metabo13030321 (PMC10055627; doi:10.3390/metabo13030321)
Supplement: Supplementary file 1 [file metabolites-13-00321-s001.zip › metabolites-2221249-supplementary.pdf]

Table of contents

**Page**	**Description**
**SupFile 1**	**Table S1**. Sample Information_Metadata
**SupFile 2**	**Table S4.** Statistical Analysis
**S4**	**Table S2.** Percentage of coefficient of coefficient of variation (% CV) of each metabolite analyzed, using a pool of samples as quality control validation sample (QC_val). CTL: control or healthy individuals; AD: adenoma patients, and CRC: colorectal cancer patients.
**S4**	**Table S3.** Percentage of coefficient of coefficient of variation (% CV) of each metabolite analyzed, using a commercial reference serum as quality control calibration sample (QC_cal). CTL: control or healthy individuals; AD: adenoma patients, and CRC: colorectal cancer patients.
**S5**	**Figure S1.** PCA-X scores plot obtained for the three studied groups (R^2 = 0.612 and Q^2 = 0.374) and colored by class of Batch 2. CTL – control; AD – adenoma, and CRC – colorectal cancer individuals. Scaling and transformation were autoscaled and logarithm.
**S5**	**Figure S2.** PCA-X scores plot obtained for the three studied groups (R^2 = 0.612, and Q^2 = 0.374) and colored by gender. 0 – female, and 1 – male individuals. Scaling and transformation were autoscaled and logarithm.
**S6**	**Figure S3.** PCA-X scores plot obtained for the three studied groups (R^2 = 0.612, and Q^2 = 0.374) and colored by fecal occult blood. Scaling and transformation were autoscaled and logarithm
**S6**	**Figure S4.** PLS-DA scores plot obtained for the three studied groups (R^2 = 0.302, and Q^2 = 0.149). CTL – control; AD – adenoma, and CRC – colorectal cancer individuals. Scaling and transformation were autoscaled and logarithm.
**S7**	**Figure S5.** OPLS-DA scores plot obtained for control individuals (CTL) and colorectal cancer patients (CRC) groups (R^2 = 0.554, and Q^2 = 0.276, CV-ANOVA *p*-value = 0.2431). CTL – control; AD – adenoma, and CRC – colorectal cancer individuals. Scaling and transformation were autoscaled and logarithm.
**S7**	**Figure S6.** OPLS-DA scores plot obtained for adenoma (AD) and colorectal cancer (CRC) groups (R^2 = 0.675, and Q^2 = 0.296, CV-ANOVA *p*-value = 0.1778). CTL – control; AD – adenoma, and CRC – colorectal

	cancer individuals. Scaling and transformation were autoscaled and logarithm.
**S8**	**Figure S7.** PCA-X scores plot obtained for the three studied groups (R^2 = 0.482, and Q^2= 0.23) when fusing Batch 2 and Batch 3 studies. CTL - control; AD – adenoma, and CRC – colorectal cancer individuals. Scaling and transformation were autoscaled and logarithm.

Table S2. Percentage of coefficient of variation (% CV) of each metabolite analyzed, using a pool of samples as quality control validation sample (QC_val). CTL: control or healthy individuals; AD: adenoma patients, and CRC: colorectal cancer patients.

	ChoE(18:1)	ChoE(18:2)	ChoE(20:4)	PE(16:0/18:1)	SM(d18:1/23:0)	SM(42:3)	TG(54:1)
**poolCTL**	10%	22%	24%	20%	9%	18%	13%
**poolAD**	7%	29%	14%	18%	12%	16%	16%
**poolCRC**	13%	21%	40%	19%	8%	11%	9%

Table S3. Percentage of coefficient of variation (% CV) of each metabolite analyzed, using a commercial reference serum as a quality control calibration sample (QC_cal). CTL: control or healthy individuals; AD: adenoma patients, and CRC: colorectal cancer patients.

	ChoE(18:1)	ChoE(18:2)	ChoE(20:4)	PE(16:0/18:1)	SM(d18:1/23:0)	SM(42:3)	TG(54:1)
**poolCTL**	10%	24%	21%	14%	11%	19%	18%
**poolAD**	8%	28%	6%	7%	11%	15%	11%
**poolCRC**	12%	22%	41%	11%	8%	12%	19%
**QC_val**	3%	5%	8%	5%	4%	5%	4%

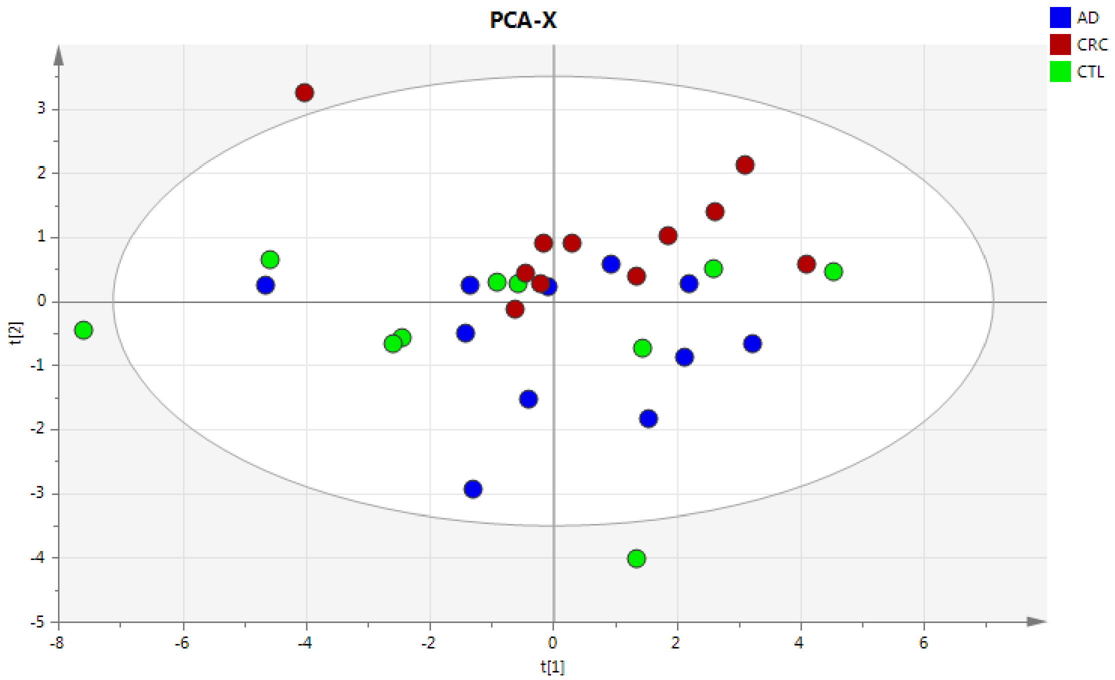

Figure S1. PCA-X scores plot obtained for the three studied groups ($R^2 = 0.612$, and $Q^2 = 0.374$) and colored by class of Batch 2. CTL - control; AD – adenoma, and CRC – colorectal cancer individuals. Scaling and transformation were autoscaled and logarithm.

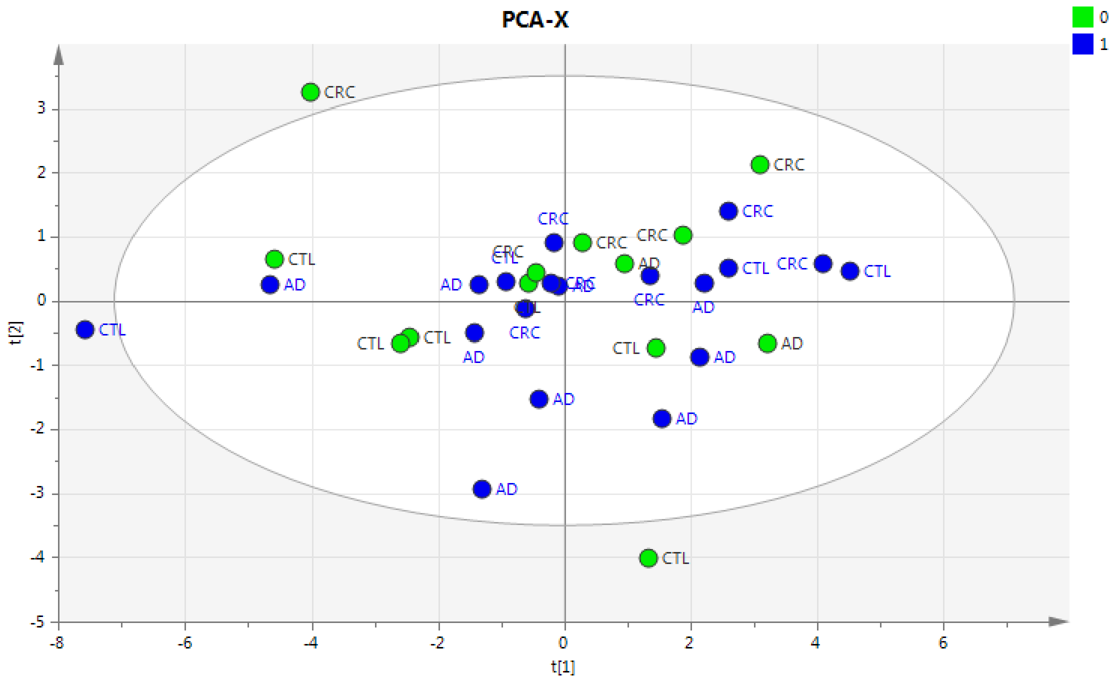

Figure S2. PCA-X scores plot obtained for the three studied groups ($R^2 = 0.612$, and $Q^2 = 0.374$) and colored by gender. 0 – female, and 1 – male individuals. Scaling and transformation were autoscaled and logarithm.

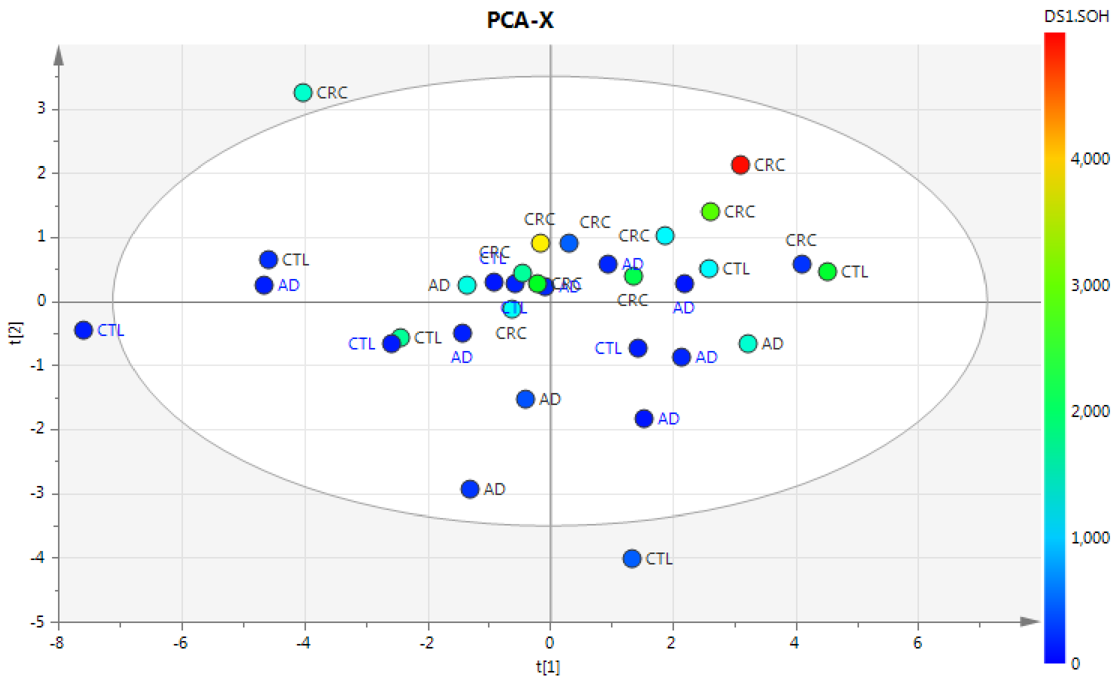

Figure S3. PCA-X scores plot obtained for the three studied groups ($R^2 = 0.612$, and $Q^2 = 0.374$) and colored by fecal occult blood. Scaling and transformation were autoscaled and logarithm.

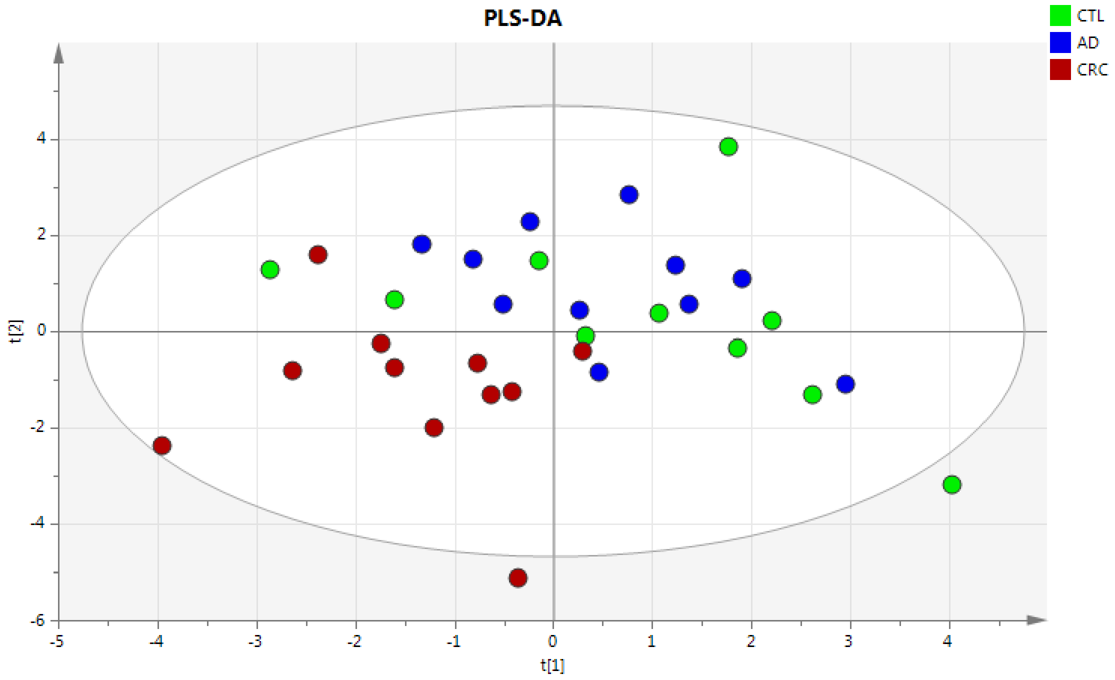

Figure S4. PLS-DA scores plot obtained for the three studied groups ($R^2 = 0.302$, and $Q^2 = 0.149$). CTL - control; AD – adenoma, and CRC – colorectal cancer individuals. Scaling and transformation were autoscaled and logarithm.

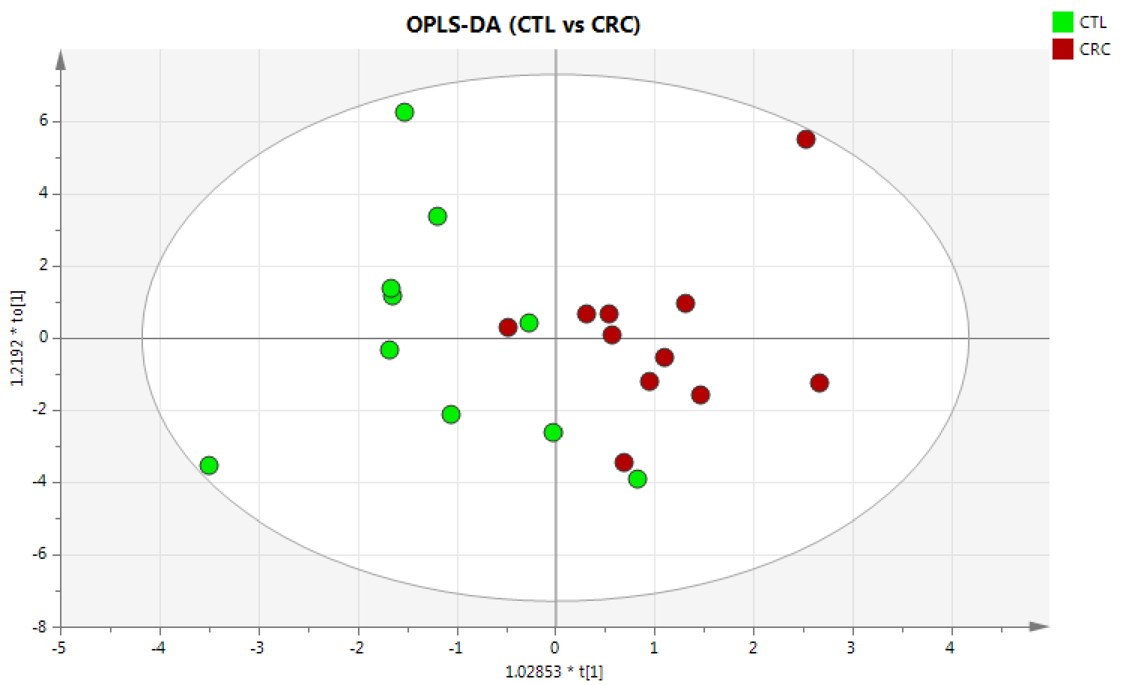

Figure S5. OPLS-DA scores plot obtained for control individuals (CTL) and colorectal cancer patients (CRC) groups ($R^2 = 0.554$, and $Q^2 = 0.276$; CV-ANOVA *p*-value = 0.2431). CTL – control individuals, and CRC – colorectal cancer patients. Scaling and transformation were autoscaled and logarithm.

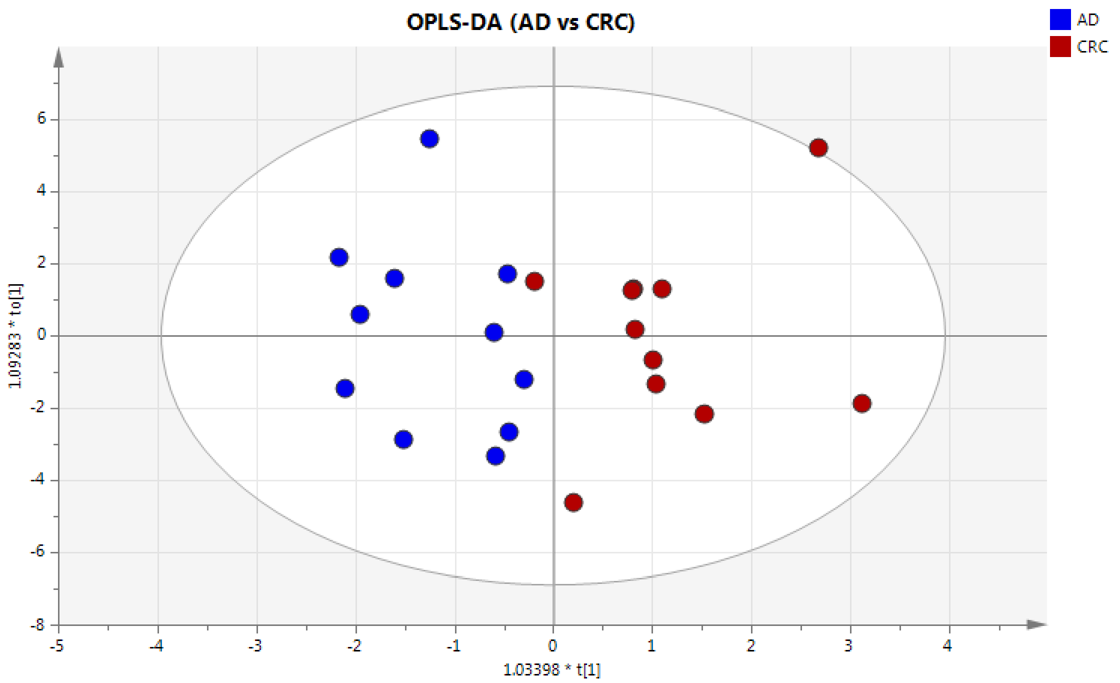

Figure S6. OPLS-DA scores plot obtained for adenocarcinoma (AS) and colorectal cancer (CRC) groups ($R^2 = 0.675$, and $Q^2 = 0.296$; CV-ANOVA *p*-value = 0.1778). AD – adenoma, and CRC – colorectal cancer patients. Scaling and transformation were autoscaled and logarithm.

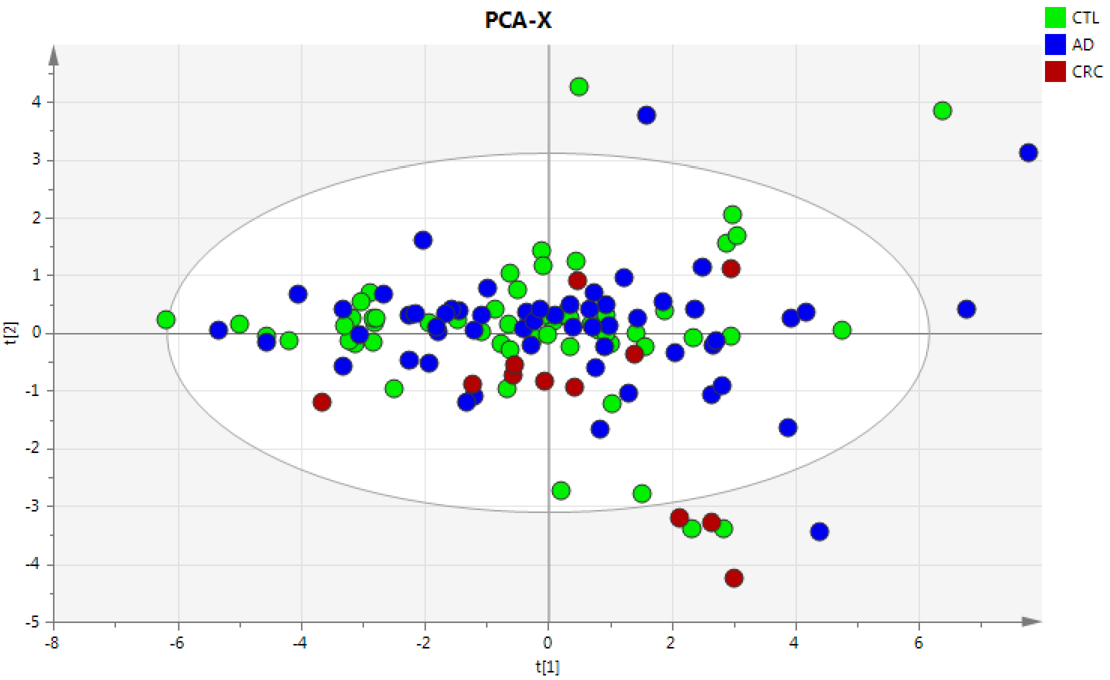

Figure S7. PCA-X scores plot obtained for the three studied groups (R^2 = 0.482, and Q^2= 0.23) when fusing Batch 2 and Batch 3 studies. CTL - control; AD – adenoma, and CRC – colorectal cancer individuals. Scaling and transformation were autoscaled and logarithm.
